# Target and reality of adjuvant endocrine therapy in postmenopausal patients with invasive breast cancer

**DOI:** 10.1038/sj.bjc.6604525

**Published:** 2008-07-29

**Authors:** U Güth, D J Huang, A Schötzau, R Zanetti-Dällenbach, W Holzgreve, J Bitzer, E Wight

**Affiliations:** 1 Department of Gynaecology and Obstetrics, University Hospital Basel (UHB), Spitalstrasse 21, Basel CH-4031, Switzerland; 2 Schötzau and Simmen Institute for Biomathematics, Malzgasse 9, Basel CH-4052, Switzerland

**Keywords:** breast cancer, adherence, compliance, aromatase inhibitor, tamoxifen, treatment

## Abstract

Previous research evaluating the use of adjuvant endocrine therapy among postmenopausal breast cancer patients showed with 15–50% wide ranges of non-adherence rates. We evaluated this issue by analysing an unselected study group comprising of 325 postmenopausal women, diagnosed from 1997 to 2003 with hormonal receptor-positive invasive breast cancer. The different clinical situations that led to the discontinuation of adjuvant endocrine therapy were clearly defined and differentiated: non-adherence was not simply the act of stopping medication, but rather the manifestation of an intentional behaviour of the patient. Of the 287 patients who initiated endocrine therapy, 191 (66.6%) fully completed this treatment. Thirty-one patients (10.8%) showed non-adherence to therapy. Patients who had follow-up with a general practitioner, rather than in an oncologic unit, were more likely to be non-adherent (*P*=0.0088). Of 25 patients who changed medication due to therapy-related adverse effects, 20 (80%) patients fully completed the therapy after drug change. In adjuvant endocrine therapy, a lowering of the non-adherence rate to 10.8%, the lowest reported in the literature, is realistic when patients are cared for by a specialised oncologic unit focusing on the individual needs of the patients.

Since the late 1990s, adjuvant systemic endocrine therapy was recommended for the vast majority of postmenopausal patients with hormonal receptor (HR)-positive breast carcinomas. The sixth St Gallen International Consensus Panel on the Treatment of Primary Breast Cancer refined these guidelines in 1998: with the exception of low-risk, node-negative patients, the panel recommended a full 5 years of tamoxifen therapy for all elderly women with HR-positive breast cancer (Goldhirsch *et al*, 1998). More recently, clinical studies demonstrated additional benefit with aromatase inhibitors in postmenopausal women, either as initial management or following a period of tamoxifen use (Coombes *et al*, 2004; Coates *et al*, 2007; Forbes *et al*, 2008).

In long-term therapy, however, there are a multitude of factors and clinical situations that prevent and threaten the completion of the targeted 5-year treatment. Patient refusal to initiate the recommended antihormonal medication and non-adherence to therapy (defined as a composite of compliance and persistence: the patient started therapy, but discontinued the planned treatment) are important, but are not the only contributors. Both factors were evaluated in several studies, in clinical trials (Fisher *et al*, 1989; Coombes *et al*, 2004; Coates *et al*, 2007; Forbes *et al*, 2008) and in clinical practise settings (Waterhouse *et al*, 1993; Demissie *et al*, 2001; Silliman *et al*, 2002; Partridge *et al*, 2003, 2008; Fink *et al*, 2004; Grunfeld *et al*, 2005; Atkins and Fallowfield, 2006; Lash *et al*, 2006; Barron *et al*, 2007; Owusu *et al*, 2008). These studies, however, evaluated only selected populations and failed to describe all the possible situations that determine compliance and adherence to therapy in everyday clinical settings.

Our study depicts the entire picture of the adjuvant treatment setting in postmenopausal women. This approach was made possible by considering the entire group of all postmenopausal patients with non-metastatic breast cancer (diagnosed and surgically treated in a 7-year period from 1997 to 2003), whose carcinomas were HR–positive, and therefore were candidates for adjuvant endocrine therapy. We define and evaluate the diverse clinical situations that hinder the realisation of the target of a complete 5-year therapy.

## Patients and methods

Data regarding all postmenopausal non-metastatic breast cancer patients, who received surgical therapy between 1997 and 2003 at the Department of Gynaecology and Obstetrics of the University Hospital Basel (Basel, Switzerland), form the basis of the current analysis. Of these 393 patients, 325 had HR-positive carcinomas (82.7%) and 68 had HR-negative tumours (17.3%). The 325 patients with HR-positive carcinomas were evaluated in this study. We had complete follow-up in 323 of these patients, whereas two patients were lost to follow-up, one after 3 months and the other after 12 months.

The following data were collected from the medical records and were available for all patients: age at initial diagnosis, tumour stage according to the American Joint Committee on Cancer (AJCC)/International Union Against Cancer (UICC) TNM Classification, histologic subtype, grading, oestrogen receptor (ER) and progesterone receptor (PR) status, surgery type, and receipt of adjuvant chemotherapy and/or radiation. HER-2/neu status was available for 273 patients (84.0%).

The treatment recommendations for all patients were based on the decision of the interdisciplinary tumour board of the University Hospital Basel. Since 1997, all HR-positive patients, with few exceptions, were recommended to have adjuvant endocrine therapy. All patients received a comprehensive consultation at the departmental oncology unit during which the treatment indication and duration, as well as the potential adverse effects, were extensively discussed.

The prescribed antihormonal agent given was recorded. To systematically evaluate the different clinical situations during the course of adjuvant endocrine therapy, we created the following subdivisions:

*(A) Patients who did not initiate therapy*

This subgroup includes the patients to whom endocrine therapy was not recommended, and the patients who refused the recommended therapy and never began treatment.

*(B) Patients who initiated therapy*

This subgroup includes the patients who completed the therapy after a 5-year course (for the patients diagnosed in 2003, we had a follow-up for at least 4 years). Patients with extended therapy >5 years were also considered as having fully completed therapy. Further subgroups must also be considered as having completed therapy, although treatment was not administered during the targeted 5 years: discontinuation due to death, as well as discontinuation due to breast cancer recurrence (local and/or distant metastases) and serious medical reasons independent from breast cancer disease and therapy-related adverse effects. We distinguished the above-mentioned subgroups from non-adherent patients who discontinued the planned mode of treatment and refused to continue further endocrine therapy.

Furthermore, we recorded any change of endocrine agents and the indication for the change: sequential therapy (switching after 2–3 years of adjuvant tamoxifen therapy to an aromatase inhibitor), extended therapy beyond 5 years of adjuvant therapy or change due to adverse effects. Lastly, we recorded the location of treatment and follow-up of the patients (our own oncology unit, external oncology unit or general practitioner).

Information concerning type and length of the medication, as well as the reasons for discontinuation, was retrospectively obtained from the medical record. Patients who had no follow-up at our institution were contacted via telephone. Afterwards, contact was made with the treating physician to confirm the patients' statements.

The study design and data collection methods were approved by the Institutional Review Board.

### Statistical analysis

To identify factors associated with treatment refusal and non-adherence, we created two univariate logistic regression models. Each model included the independent variables: year of the initial diagnosis, patient's age, primary surgical therapy, tumour stage, receipt of previous chemotherapy and/or postoperative radiation, and location of follow-up (the latter only in the non-adherence model). Odds ratios (ORs) with corresponding 95% confidence intervals (CIs) were reported; a *P*-value less than 0.05 was considered significant. Statistical analyses were performed with R Development Core Team Software (version 2.5.0, Vienna, Austria).

## Results

The clinicopathologic, treatment and follow-up characteristics of the 325 patients in the study are summarised in Tables 1, 2 and 3.

*(A) Patients who did not initiate endocrine therapy (n*=*38)*

In 10 cases, despite HR-positive carcinoma, a recommendation for endocrine therapy was not made. The reasons for this included a low-risk constellation (pT1a/b N0, favourable grading) and/or advanced age with considerable comorbidity. Twenty-eight patients (8.9%) refused the recommended endocrine therapy after extensive counselling and never began this treatment. Univariate analysis revealed that older patients, those who were initially diagnosed at the beginning of the study period and those who did not receive adjuvant chemotherapy were more likely to refuse therapy (advanced age: *P*=0.019; study period: 1997–2000 *vs* 2001–2003: *P*=0.0070; no chemotherapy: *P*<0.0001;Table 4). Additionally, patients who had a favourable disease stage (stage I) tended to refuse the recommended therapy (*P*=0.0546).

*(B) Patients who initiated endocrine therapy (n*=*287)*

The initial endocrine agents prescribed are listed in Table 2. Forty-five patients were treated within the randomised, phase III, double-blind Breast International Group (BIG) 1-98 four-arm study (letrozole or tamoxifen as monotherapy or sequential therapy) (Coates *et al*, 2007). The medication was unblinded in 25 of these patients. Even when the study drug had been unblinded, these cases were listed under the category ‘study medication (BIG 1-98)’. Of the 45 BIG 1-98 study patients, 30 (66.7%) had completed 5 years of treatment according to the study guidelines. Of the 14 patients who prematurely discontinued their randomly assigned trial medication for reasons other than disease recurrence, 8 women fully completed the endocrine therapy over a 5-year course outside of the trial (57.3%).

In 50 patients, there was a change of the antihormonal agent prescribed within the planned 5-year period. Of these, four patients changed their medication twice and one patient three times. Twenty-five of the 50 patients changed their medication due to adverse effects of the endocrine therapy (three changed the medication twice and one three times). Of these, 20 patients (80%) fully completed the therapy after drug change.

The therapy course, reported in Tables 3 and 5, was as follows:

Of the 287 patients who initiated endocrine therapy, 191 (66.6%) fully completed the targeted therapy (four patients interrupted therapy for up to 3 months, another for 5 months). Eleven patients (3.8%) discontinued the therapy due to death, whereas 43 patients (15.0%) ceased therapy due to breast cancer recurrence. In nine cases (3.1%), the therapy was discontinued by the physician due to serious medical reasons independent from breast cancer disease and therapy-related adverse effects (advanced age/dementia/need for nursing home care, *n*=5; incurable malignancy other than breast cancer, *n*=3; irreversible coma following severe head trauma, *n*=1).

*Non-adherence*: Thirty-one patients (10.8%) chose independently to end the therapy. The main reasons for this given by the patients are listed in Table 5. In the seven variables used in our univariate analyses, location of follow-up was the only significant predictor, showing that patients who did not have follow-up in an oncologic unit, but rather with a general practitioner, were more likely to be non-adherent to therapy (*P*=0.0088; Table 4).

*Location of follow-up*: Of the 289 patients who received adjuvant endocrine therapy, 215 (74.4%) were treated in an oncology unit; of these, 194 patients in the internal oncology unit (67.1%) and 21 (7.3%) in an external oncologic unit. Seventy-two patients (24.9) had further follow-up and treatment through a general practitioner. Two patients (0.7%) were lost to follow-up, and the location of the oncologic care was unknown.

## Discussion

Although several studies performed in clinical practise settings (Waterhouse *et al*, 1993; Demissie *et al*, 2001; Silliman *et al*, 2002; Partridge *et al*, 2003, 2008; Fink *et al*, 2004; Grunfeld *et al*, 2005; Atkins and Fallowfield, 2006; Lash *et al*, 2006; Barron *et al*, 2007; Owusu *et al*, 2008) and clinical trials (Fisher *et al*, 1989; Coombes *et al*, 2004; Coates *et al*, 2007; Forbes *et al*, 2008) have addressed non-adherence to endocrine therapy for breast cancer, the conclusions that can be drawn from the currently available evidence are significantly limited. The reported rates of non-adherent patients in adjuvant endocrine therapy of breast cancer range considerably from 15 to 50%. The great variability of the data can only be adequately interpreted when the basic methods of each study are closely analysed. Within the current literature, there exist major non-uniformities and shortcomings, which make useful insights for a current general population difficult. Some of the critical points include

- All studies exclusively analysed selected study cohorts.
- Some studies used insurance claims and public health data (Partridge *et al*, 2003, 2008; Barron *et al*, 2007). However, analyses of public databases, which record patient data only to the extent that was necessary for administrative statistical purposes, are not always able to provide information concerning the often very individual clinical situations and reasons that lead to the cessation of medication.
- In some studies, it must be assumed that also patients who never took the medication, that is patients who received the prescription but refused therapy from beginning, were also considered (Partridge *et al*, 2003, 2008; Barron *et al*, 2007).
- Most clinical practise studies only analysed a predominantly geriatric study group (Silliman *et al*, 2002; Partridge *et al*, 2003; Fink *et al*, 2004; Lash *et al*, 2006; Barron *et al*, 2007; Owusu *et al*, 2008).
- The data are difficult to compare due to variable observation periods, which included 17 months (Silliman *et al*, 2002), over 3 (Demissie *et al*, 2001; Barron *et al*, 2007; Partridge *et al*, 2008), 4 (Partridge *et al*, 2003), and up to 5 (Fink *et al*, 2004; Lash *et al*, 2006; Owusu *et al*, 2008) years.
- Large part of the published studies are not applicable today, since initial diagnosis and the start of adjuvant treatment took place in the 1990s when the indication for therapy was usually limited to patients with stage II/III disease (Demissie *et al*, 2001; Silliman *et al*, 2002; Partridge *et al*, 2003; Fink *et al*, 2004; Lash *et al*, 2006; Owusu *et al*, 2008). In contrast, endocrine therapy is recommended for nearly all HR-positive breast cancer patients according to the most current guidelines (Goldhirsch *et al*, 2007). Only two studies have reported results from data obtained in a contemporary study period after 2001 (Barron *et al*, 2007; Partridge *et al*, 2008).

It should be the goal of studies concerning the use of endocrine therapy to identify the rate of patient non-compliance and non-adherence and to evaluate the characteristics of these patients. A certain proportion of these patients may be potentially motivated to initiate and to maintain therapy by more intensive care and improved counselling. In our view, non-adherence is not simply the act of stopping medication, but rather the manifestation of an intentional behaviour. The reasons for non-adherence, such as distressing adverse effects, inadequate clarification of the benefits of therapy, and fear and mistrust of the agent prescribed, can be elucidated in most cases. An important aspect of non-adherence to treatment is the ability of physician to intervene and change the attitude that led to the discontinuation. To separately analyse the subgroup of non-adherent patients, the clinical situations that result in a discontinuation of medication must be clearly defined. Particularly, patients who refused the recommended therapy or were non-adherent (whose attitude and behaviour may be potentially influenced) and those whose therapy had to be stopped due to breast cancer recurrence or other serious medical reasons (that is, discontinuation of the therapy was unavoidable) must not be analysed in one collective study group.

A realisation of this goal requires a careful and detailed clinical follow-up of the study group and a clear discrimination between the terms and reasons behind ‘discontinuation’ and ‘non-adherence’. The currently available studies from clinical practise settings have not been able to accomplish this. In most of these studies, patients who discontinued therapy, regardless of the reason for cessation, were categorised in a single group, and the terms ‘discontinuation’ and ‘non-adherence’ were used synonymously (Waterhouse *et al*, 1993; Silliman *et al*, 2002; Partridge *et al*, 2003, 2008; Fink *et al*, 2004; Grunfeld *et al*, 2005; Atkins and Fallowfield, 2006; Lash *et al*, 2006; Barron *et al*, 2007; Owusu *et al*, 2008).

Large clinical trials, with their extensive case documentation requirements, also cannot provide exact information regarding adherence to endocrine therapy in the general setting, as the readiness to participate in a trial already creates a certain selection bias. In trials that compared tamoxifen with an aromatase inhibitor, the rates of non-adherence are clearly lower than those reported in clinical practise studies, and within those trials, the variabilities in rates were minor (tamoxifen: 11–13%, aromatase inhibitors: ∼12%) (Coombes *et al*, 2004; Coates *et al*, 2007; Forbes *et al*, 2008). The NSABP B-14 trial, conducted in the 1980s, revealed a non-adherence rate with tamoxifen of 16.8%; remarkably, non-adherence to placebo was also comparatively high (14.9%) (Fisher *et al*, 1989). Non-adherence data from clinical trials may not necessarily be transferable to general practise, as the withdrawal of study medication does not imply the stop of endocrine therapy. In our analysis, 8 out of 14 patients who stopped the study medication in the BIG 1-98 trial due to other reasons than breast cancer recurrence continued and completed endocrine therapy outside of the trial.

The conclusions of all currently available studies can only give more or less reliable information regarding the use of endocrine therapy for postmenopausal breast cancer in clinical practise today. In our study, we avoided many of the above-mentioned methodological problems:

- We formulated our study group following current guidelines, which indicate endocrine therapy based on menopausal status, and not on a defined age.
- We utilised a more population-based approach by analysing and following all surgically treated postmenopausal patients in a 7-year period; through this, we were able to minimise selection bias.
- The data reflect the current situation, as a period was analysed in which the currently valid guidelines of treatment recommendations (Goldhirsch *et al*, 2007) were active.
- We provided careful and detailed clinical follow-up with clear differentiation of the situations that led to discontinuation of therapy.
- We used a clear definition of the term ‘non-adherence’.

We did not differentiate between antihormonal agents prescribed in our study based on the finding from clinical trials that adherence to aromatase inhibitors and tamoxifen did not differ significantly (Chlebowski and Geller, 2006).

The rate of non-adherence to adjuvant endocrine therapy in our study was with 10.8% considerably lower than that reported in most other clinical practise settings (21–50%) (Partridge *et al*, 2003, 2008; Fink *et al*, 2004; Lash *et al*, 2006; Barron *et al*, 2007; Owusu *et al*, 2008); these studies, however, were plagued by the previously mentioned methodological weaknesses. With a non-adherence rate of 15% after a 3-year observation period, Demissie *et al* (2001) had findings most similar to ours. Not surprisingly, they avoided the major methodical shortcoming of other studies, in that they excluded patients who stopped therapy due to breast cancer recurrence from the group of non-adherent patients. When compared to the non-adherence rates reported in clinical trials (approximately 12%; Coombes *et al*, 2004; Coates *et al*, 2007; Forbes *et al*, 2008), our results appear realistic.

Several studies performed in a clinical practise setting demonstrated that adherence to endocrine therapy was influenced by certain factors such as age, adverse effects, patient beliefs about the risks and benefits of tamoxifen use, history of medication and good patient–doctor relationship (Chlebowski and Geller, 2006). Some of these factors may be influenced by the type of care and expertise of the responsible physician. Our study shows that care in an oncologic unit is significantly associated with higher adherence to therapy (non-adherence in the subgroup of patients who had follow-up in an oncologic unit was even as low as 7.4%). The finding that care in an oncologic unit is associated with higher adherence may not necessarily be a reason for optimism. With more and more numbers of cancer patients being treated with oral agents and limitations on specialists' time, a better strategy might be to develop improved collaboration with primary care physicians.

As most of the patients of our study were treated at the oncology unit of our department, the favourable non-adherence rates are probably associated with the fact that all practitioners received a targeted education in the techniques of patient-centred communication through the departmental division of psychosomatic medicine. These communication skills were applied in the follow-up of oncologic patients, in particular regarding the dialogue concerning possible therapy-related side effects and the importance of compliance and adherence to therapy (Mallinger *et al*, 2005; Stewart *et al*, 2007). Although endocrine therapy for postmenopausal breast cancer is generally well tolerated and the majority of adverse effects are mild to moderate, there are a certain number of patients who experience these as distressing (above all, hot flushes, musculoskeletal complaints/arthralgia and vaginal dryness). These effects should be carefully evaluated and regarded seriously in the follow-up appointments making the patients feel that their complaints are taken earnestly. As stated by other authors, a change in therapy may be an effective strategy to improve patient adherence (Hadji, 2008). In our study, 80% of the patients who required a change of the prescribed agent due to adverse effects could fully complete endocrine therapy in the course of time.

The limitations of our retrospective study, however, must be considered. First, our study relies on information obtained by patients' self-report of adherence. It is possible that in some cases, the patients who reported continuing to take medication had indeed stopped taking it and just gave a socially acceptable answer. Furthermore, we defined non-adherence as an intentional behaviour. Atkins and Fallowfield (2006) demonstrated a high percentage of 55% of non-adherent patients with the vast majority of women reporting a non-intentional non-adherence, that is they often just forgot to take their medication. On the other hand, we think that it is unlikely that women would report that they had stopped taking medication when in fact they had not. In this context, the findings of Waterhouse *et al* (1993) must also be considered; they examined the influence of methodology on the categorisation of adherence to tamoxifen therapy and found that self-reported adherence fairly consistently underestimates non-adherence as determined by more objective measures, such as pill count and microelectronic monitoring. A second caveat may be that our study comes from a single region of a small country with a high socioeconomic status. All inhabitants of Switzerland have universal access to health care and free access to all prescribed and approved drugs. These facts must be considered while interpreting our relatively low non-adherence rate.

Our data show that, when compared with other studies, low non-adherence rates can be realistically achieved. In the future, multifactorial approaches should be further analysed and refined with the goal of further improving compliance and adherence, and by doing so, the outcome of breast cancer patients (Chlebowski and Geller, 2006).

## Figures and Tables

**Table 1:** Clinicopathologic and treatment characteristics of 325 postmenopausal women with hormonal receptor-positive breast cancer

**Variable**	**Number (%)**
Age (years)	
Mean	67.3
Range	47–95
⩽60 years	100 (30.8)
61–75 years	152 (46.8)
>75 years	73 (22.4)
	
*Hormonal receptor status*	
ER+ PR+	231 (71.1)
ER+ PR−	89 (27.4)
ER− PR+	5 (1.5)
	
*Grading*	
G1	74 (22.8)
G2	166 (51.1)
G3	85 (26.1)
	
*HER-2 neu status*	
Known	273 (84.0)
Positive	42 (15.4)
	
*Histologic subtype*	
Ductal invasive	233 (71.7)
Lobular invasive	62 (19.1)
Rare types	30 (9.2)
	
*AJCC/UICC stage* a	
I	144 (44.3)
IIA	134 (41.2)
III	47 (14.5)
	
*Type of surgery*	
Mastectomy+ALND	101 (31.1)
Mastectomy+SLND	4 (1.2)
BCT+ALND	143 (44.0)
BCT+SLND	39 (12.0)
Mastectomy only	23 (7.1)
Tumorectomy only	15 (4.6)
	
*Systemic treatment, others than adjuvant endocrine therapy*	
Previous adjuvant chemotherapy	49 (15.1)
Neoadjuvant chemotherapy	4 (1.2)
Neoadjuvant endocrine therapy	2 (0.6)
Adjuvant treatment with trastuzumab	2 (0.6)
Adjuvant radiotherapy	196 (60.3)

AJCC=American Joint Committee on Cancer; ALND=auxiliary lymph node dissection; BCT=breast conserving therapy; ER=estrogen receptor; PR=progesterone receptor; SLND=sentinel lymph node dissection; UICC=International Union Against Cancer.

a In six patients, where neoadjuvant therapy was performed, the ypT and ypN status were used for stage grouping.

**Table 2:** Endocrine therapy regimen

	**Number (%)**
*Initial agent prescribed*	
Tamoxifen	206 (63.4)
Anastrozol	18 (5.5)
Letrozole	18 (5.5)
Study medication (BIG 1-98 trial)	45 (13.8)
	
*Change of the agent prescribed*	
Owing to adverse effects	30
Sequential therapy (switching after 2–3 years of adjuvant tamoxifen therapy^a^	26
Extended therapy beyond 5 years of adjuvant tamoxifen therapy	23
	
*Change of the agent prescribed within the 5-year period of treatment (excluding patients with change of the regimen due to extended therapy)*	
Study medication (BIG 1-98) → tamoxifen	4 (7.1)
Study medication (BIG 1-98) → aromatase inhibitor	6 (10.7)
Tamoxifen → aromatase inhibitor	37 (66.1)
Aromatase inhibitor → tamoxifen	3 (5.4)
Aromatase inhibitor → aromatase inhibitor	6 (10.7)
	
*Change of the agent prescribed within the 5-year period of treatment due to adverse effects*	
Study medication (BIG 1-98) → tamoxifen	4 (13.3)
Study medication (BIG 1-98) → aromatase inhibitor	4 (13.3)
Tamoxifen → aromatase inhibitor	14 (46.7)
Aromatase inhibitor → tamoxifen	4 (13.3)
Aromatase inhibitor → aromatase inhibitor	4 (13.3)

BIG=Breast International Group.

a Owing to medication blinding, switching endocrine therapy within the BIG 1-98 trial was not considered.

**Table 3:** Course of endocrine therapy

	**Number (%)**	**Mean age (range)**	**Median duration of therapy (months)**
*Entire study group*	325		
No endocrine therapy recommended	10 (3.1)	76.8 (51–95)	
			
*Patients where endocrine therapy was recommended*	315 (100)		
Patients refused to initiate therapy	28 (8.9)	70.4 (51–89)	
			
*Patients who initiated endocrine therapy*	287 (100)		
Therapy fully completed	191 (66.6)	65.0 (47–85)	
Therapy discontinued due to death (i.e., completed)	11 (3.8)	81.4 (69–88)	12 (4–48)
Therapy discontinued due to breast cancer recurrence (i.e., completed)	43 (15.0)	67.1 (48–86)	28 (4–58)
Therapy stopped due to medical reasons independent from breast cancer and adverse effects from the endocrine therapy	9 (3.1)	76.0 (52–91)	28 (5–52)
Non-adherence	31 (10.8)	67.3 (51–85)	19 (1–50)
Lost to follow-up	2 (0.7)		

**Table 4:** Univariate relationships between potential predictors and refusal of recommended endocrine therapy (A) and non-adherence to therapy (B)

**Variable**	**Odds ratio**	**95% CI**	***P*-value**
*(A) Refusal of recommended therapy (n*=*28)*			
*Year of the initial diagnosis*			
2001–2003	Reference		
1997–2000	3.07	1.36–6.94	0.0070
Advanced age	1.54^a^	1.07–2.21	0.0190
			
*TNM disease stage*^b^			
Stage II/III	Reference		
Stage I	2.12	0.99–4.57	0.0546
			
*Primary surgical therapy*			
BCT	Reference		
Mastectomy	1.31	0.59–2.91	0.5010
No previous chemotherapy	38.83	11.32–133.16	<0.0001
Postoperative radiation	1.82	0.85–3.87	0.1211
			
*(B) Non-adherence (n*=*31)*			
*Year of the initial diagnosis*			
2001–2003	Reference		
1997–2000	1.10	0.52–2.32	0.8115
Advanced age	1.09^a^	0.75–1.60	0.6501
			
*TNM disease stage1*			
Stage II/III	Reference		
Stage I	1.37	0.65–2.90	0.4044
			
*Primary surgical therapy*			
BCT	Reference		
Mastectomy	1.43	0.65–3.17	0.3748
No previous chemotherapy	2.23	0.65–7.64	0.2013
Postoperative radiation	1.82	0.86–3.84	0.1186
			
*Location of follow up*			
Oncologic unit	Reference		
General practitioner	2.78	1.29–5.98	0.0088

BCT=breast conserving therapy.

a Odds ratio is expressed as the ratio of an increase within 10 years.

b AJCC (American Joint Committee on Cancer)/UICC (International Union Against Cancer) TNM Classification.

**Table 5:** Non-adherence to endocrine therapy: main reasons for and period of discontinuation in 31 non-adherent patients

	**Number (%)**
*Main reasons for discontinuation*	
Without reporting complaints	11 (35.5)
Intolerance, general discomfort and malaise	9 (29.0)
Hot flushes	4 (12.9)
Dermatologic symptoms/hair loss	3 (9.7)
Visual disturbances	1 (3.2)
Alcohol dependency or psychiatric disease	3 (9.7)
	
*Period of discontinuation (year of therapy)*	
First year	12 (38.7)
Second year	6 (19.4)
Third year	9 (29.0)
Fourth year	3 (9.7)
Fifth year	1 (3.2)
